# Systems-based approaches to unravel multi-species microbial community functioning

**DOI:** 10.1016/j.csbj.2014.11.009

**Published:** 2014-12-03

**Authors:** Florence Abram

**Affiliations:** Functional Environmental Microbiology, School of Natural Sciences, National University of Ireland Galway, University Road, Galway, Ireland

**Keywords:** Systems biology, Metagenomics, Metatranscriptomics, Metaproteomics, Metabolomics, Microbial ecology

## Abstract

Some of the most transformative discoveries promising to enable the resolution of this century's grand societal challenges will most likely arise from environmental science and particularly environmental microbiology and biotechnology. Understanding how microbes interact in situ, and how microbial communities respond to environmental changes remains an enormous challenge for science. Systems biology offers a powerful experimental strategy to tackle the exciting task of deciphering microbial interactions. In this framework, entire microbial communities are considered as metaorganisms and each level of biological information (DNA, RNA, proteins and metabolites) is investigated along with in situ environmental characteristics. In this way, systems biology can help unravel the interactions between the different parts of an ecosystem ultimately responsible for its emergent properties. Indeed each level of biological information provides a different level of characterisation of the microbial communities. Metagenomics, metatranscriptomics, metaproteomics, metabolomics and SIP-omics can be employed to investigate collectively microbial community structure, potential, function, activity and interactions. Omics approaches are enabled by high-throughput 21st century technologies and this review will discuss how their implementation has revolutionised our understanding of microbial communities.

## 1 Introduction

**Fig. 1:**
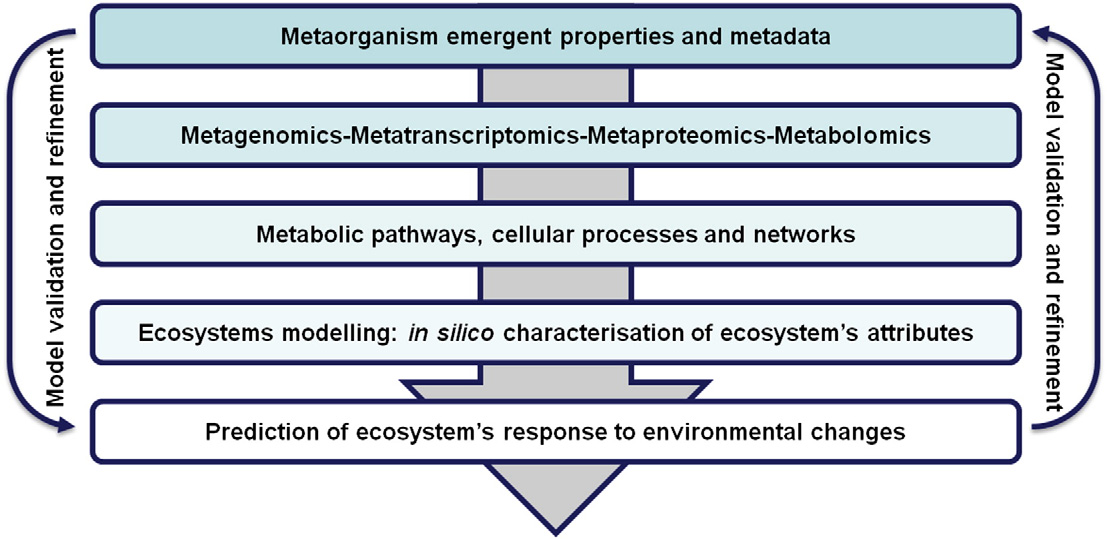
Systems biology. Integrated analysis of metadata with omics datasets provides access to the metabolic pathways, cellular processes and networks occurring in situ. The extensive datasets generated are then used to build models for the prediction of an ecosystem's response to environmental cues.

Microorganisms make up the main portion of biomass on Earth and are ubiquitous within the environment. In situ, they coexist in mixed microbial communities whose concerted actions greatly contribute to sustaining life on our planet. Microorganisms are indeed the main drivers of biogeochemical cycles and as such ensure the recycling of essential organic elements like carbon and nitrogen. In addition, microbial communities interact with plant and animal hosts, and in the context of human biology, our microbiome is now considered to be our last organ [1]. Understanding how microbes interact in situ and how microbial communities respond to environmental changes has been identified as one of the major challenges for the coming years with relevance to evolution, human health, environmental health, synthetic biology, renewable energy and biotechnology [2]. To tackle the exciting task of deciphering microbial interactions, systems biology approaches constitute an ideal experimental strategy (Fig. 1). By considering microbial communities as metaorganisms and investigating all the levels of biological information (DNA, RNA, proteins and metabolites) together with the metadata characteristic of the environmental conditions in situ, systems biology can study the interactions between the different parts of complex ecosystems responsible for their emergent properties. The success of systems biology is strongly dependent on the true integration of experimental observations and the development of mathematical models, which require iterative validation and refinement.

**Fig. 2:**
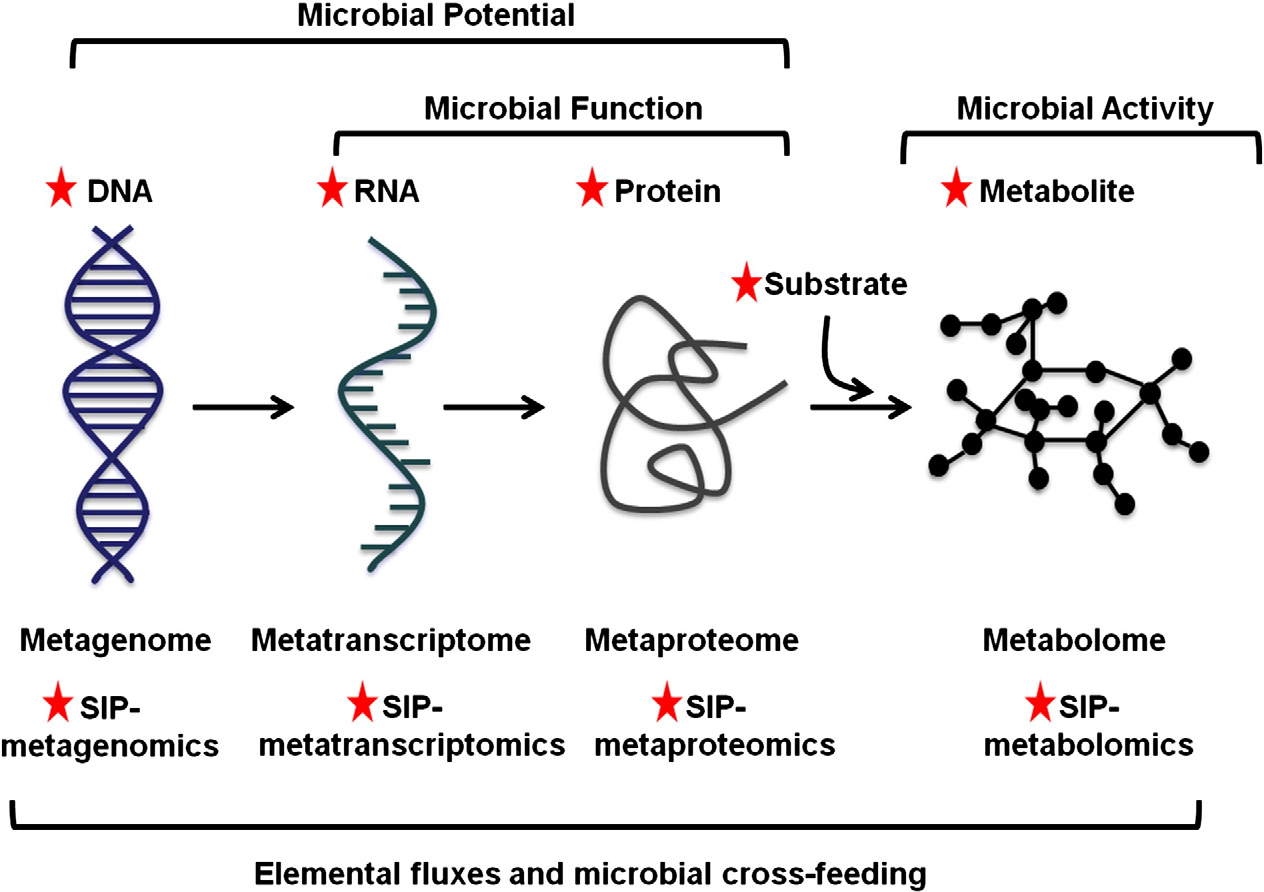
Overview of multi-omics approach. Each level of information (DNA, RNA, proteins and metabolites) provides a different level of characterisation of the microbial community. SIP stands for stable isotope probing. SIP-omics provide insights into elemental fluxes and microbial cross-feeding.

Systems biology offers a holistic approach for the characterisation of microbial communities. In such experimental designs metagenomics, metatranscriptomics, metaproteomics and metabolomics are typically employed. Each level of biological information provides a different level of characterisation of the metaorganisms (Fig. 2). The metagenome informs on the potential of microbial communities by providing insights into the genes that could possibly be expressed by the metaorganism. The metatranscriptome, including messenger and non-coding RNAs, provides some information about the regulatory networks and gene expression at the time of sampling. Therefore, together with the metaproteome, the metatranscriptome informs on the functionality of microbial communities. Furthermore, the metaproteome also gives access to regulatory networks (within, and between, cells) and, together with the metabolome provides some strong insights into microbial activities. Importantly, the co-extraction of DNA, RNA, proteins and metabolites [3] enables the generation of rigorous interrelated datasets. Each of the omics techniques has inherent bottlenecks, such as metagenome annotation, metatranscriptome assembly, or protein and metabolite identification. These bottlenecks however can be largely overcome by generating integrated datasets whereby the detection of RNA transcripts and amino acids can guide the process of metagenome annotation [4], [5]. This, in turn, can radically facilitate metatranscriptome assembly, while increasing significantly protein identification rates [6]. Meanwhile, however, metabolomics remains a complex technology. Untargeted experimental strategies are typically limited by the low number of metabolites identified. Indeed, while DNA and RNA are composed of nucleotides and proteins composed of amino acids, metabolites do not share any common characteristics making their systematic identification challenging. In addition, metabolite databases, containing mass spectra or NMR spectra (generated by mass spectrometry or nuclear magnetic resonance, respectively), are still relatively poorly populated compared to gene or protein databases. Nevertheless, metabolite databases are constantly growing and targeted metabolite identification can be guided by protein detection. Even though metabolite detection ultimately correlates with microbial activity, metabolite production in mixed populations cannot be easily linked to any specific microbial identity. Besides, metabolomics offers a limited level of information regarding the connectivity of metabolic pathways [7]. However the combination of isotope labelling, such as ^13^C and ^15^N, with omics (SIP-omics) can provide insights into the carbon and nitrogen fluxes in microbial communities and inform on microbial interrelationships. Overall omics datasets encompassing metagenomics, metatranscriptomics, metaproteomics, metabolomics and SIP-omics have the potential to provide unprecedented access to the functioning of ecosystems. For the purpose of this review, the advancement of each omics technology will be discussed.

## 2 Metagenomics: Microbial potential

Metagenomics is employed to determine the sequences from DNA directly extracted from environmental samples. This high-throughput technology, which overcomes the well-known culture-based-method biases, has transformed our understanding of microbial ecosystems in terms of diversity, population dynamics and potential. Commonly, metagenomic studies initially conduct 16S and 18S rRNA surveys (at the DNA level) to examine microbial diversity and community composition while informing on the sequencing depth required to access high levels of metagenome coverage [8], [9], [10], [11]. The resulting amplicon sequences, typically generated using Illumina or pyrosequencing platforms, are subjected to quality filtering before taxonomic assignment is performed commonly using computational tools such as QIIME and mothur [12], [13]. These data can then be used to calculate sample diversity and microbial community distance metrics in the context of comparative investigations. In addition, correlations between species and metadata can be uncovered when the microbial communities are analysed under different environmental conditions [14]. While small subunit (SSU) rRNA profiling, at the DNA level, can provide insights into community structure, the potential, flexibility and robustness of an ecosystem can only be investigated with the elucidation of deep metagenomes. A recent interesting development, however, in the exploitation of SSU rRNA data has been brought about with the introduction of PICRUSt, a computational tool to predict the functional profile of microbial communities based on gene marker surveys and the availability of reference genome databases [15]. Different sequencing platforms can be employed for metagenomics [16], and commonly metagenome sequences are composed of short-length reads, which render the process of assembly and annotation particularly challenging. In order to assemble and recover single genomes from metagenomic data, sequences are classified into discrete clusters commonly referred to as bins. Binning algorithms have been specifically developed for metagenomic sequence read assembly; examples of these include Meta-IDBA [17], AbundanceBin [18], MetaVelvet [19] and Metacluster [20], [21], [22]. Further binning strategies can then be employed to retrieve single genomes from the fragmented assembled contigs. One of the most widely used binning approaches to do this relies on emergent self-organising maps (ESOMs; 23). ESOMs can be based for example on tetranucleotide frequency distribution [24] or time series abundance profile [25]. In both contexts, individual bins are commonly selected manually from graphical outputs. To circumvent this, novel automated binning algorithms have been recently developed to recover genomes from fragmented assembled metagenomic contigs (MaxBin, MetaBAT and CONCOCT; 23,26,27). Computational tools for metagenomic annotation are also widely available such as MG-RAST and RAMMCAP [28], [29]. Obtaining meaningful functional information from metagenomic datasets can be very difficult and particularly costly in term of computational process time. This can partly be attributed to the large proportion of uncharacterised taxa prevailing in many environments. In order to address this issue, a novel manually curated database was built, FOAM, which has been demonstrated to screen metagenomic datasets for functional assignments with higher sensitivity and 80 times faster than BLAST [30]. Depending on the research question and the motivation for conducting metagenomics, assembly might not always be required. Indeed, in order to explore the metabolic potential of a microbial community, Abubucker et al. (2010) developed a computational pipeline (HUMAnN) to determine the relative abundance of gene families and metabolic pathways from short-read sequences characteristic of metagenomic datasets [31]. Similarly, Rooijers et al. (2011) designed an iterative computational workflow using raw metagenomic sequences to mine metaproteomes [32]. These two pipelines [31], [32], however, have been developed for the human microbiome and rely heavily on the availability of numerous robustly annotated genomes from relevant single microorganism. Predictive modelling approaches, such as PRMT (Predictive Relative Metabolic Turnover; 33) have been recently designed to explore multi-species community functioning in the context of metagenomics. PRMT uses metagenomic information to predict metabolite environmental matrices and generate PRMT scores. Correlations between these scores and relative phylogenetic abundance can then be investigated to infer potential metabolic role of specific taxa within an ecosystem, therefore providing a useful strategy to access community functioning from metagenomic data [33]. Metagenomics is a powerful tool to identify and in some instances isolate novel microorganisms and help uncover the distribution of metabolic capacities across the tree of life. For example, the analysis of acid mine drainage metagenomes revealed the presence of a unique *nif* operon, which led to the isolation of the only nitrogen fixer from the bacterial community by cultivating the acid mine drainage biofilm in the absence of nitrogen [34]. Recently, 12 bacterial near complete genomes were reconstructed from activated sludge metagenomic datasets [35]. These included rare, uncultured species with relative abundance as low as 0.06%, highlighting the power of metagenomics to uncover novel microorganisms [35]. Similarly, metagenomics from a premature infant gut microbiota led to the recovery of 11 near complete genomes [36]. Amongst these, the first genome of a medically relevant species, *Varibaculum cambriense*, could be reconstructed. Genomic-based metabolic prediction of *V. cambriense* unveiled the metabolic versatility of this bacterium in terms of carbon sources and electron acceptors during anaerobic respiration [36]. In addition, the dataset indicated a possible metabolic exchange between *V. cambriense* and the rest of the microbial community. While *V. cambriense* has the ability to produce nitrite, which could be further metabolised by other species, the microorganism could be dependent on the community for its source of trehalose [36]. Metagenomics of sediment samples from a site adjacent to the Colorado River (US) revealed a surprising phylogenetic diversity and novelty coupled with metabolic flexibility [37]. The microbial communities displayed a high level of evenness with no single organism accounting for over 1% of the communities. The most abundant species in deeper sediments, RBG-1, was found to represent a new phylum. The genome of RBG-1 was recovered from the metagenomic dataset and counted over 1900 protein-encoding genes [37]. Genomic-based metabolic profile reconstruction of RBG-1 highlighted its potential role in metal biogeochemistry with the capacity of iron cycling both under aerobic and anaerobic environmental conditions [37]. Metagenomic datasets from the same site were further mined to investigate the metabolic diversity of the *Choloroflexi* phylum in sediments [38]. *Choloroflexi* were found to be metabolically flexible with the ability to adapt to varying redox conditions. They were predicted to play a role in carbon cycling being able to degrade plant material such as cellulose [38]. In addition, known pathways previously not associated with this phylum were found to be encoded in the newly reconstructed genomes recovered from metagenomic datasets [38]. After discovering that thawing permafrost was commonly dominated by a single archaeal phylotype with no cultured representative, as indicated by SSU rRNA profiling from DNA samples, Mondav et al. (2014) recovered its genome from a metagenomic dataset in order to assess its metabolic capacity [39]. This novel archaea was found to be present in 33 locations widely geographically distributed and dominant in some cases accounting for up to 75% of detected archaeal sequences [39]. Metabolic reconstruction of this archaea indicated its ability to perform hydrogenotrophic methanogenesis. This was confirmed in situ by metaproteomics, and conferred a significant role to the novel methanogen in global methane production [39]. This illustrates how metagenomics can help develop biological hypotheses that can be further tested employing other omics. An important pitfall of metagenomics and its interpretation, when used in isolation, is the inherent assumption that microorganisms have the same potential and therefore perform the same function regardless of their environment. Freilich et al. (2011), together with similar work [41], [42], [43], could demonstrate that microbial interactions can be manipulated through changes in environmental conditions [40], which cannot be easily accounted for when analysing metagenomic datasets. Therefore to embrace the full potential of metagenomics, and particularly to test the derived biological hypotheses, the combination with other omics is required.

## 3 Metatranscriptomics: Microbial potential function

While metagenomics informs on the genes present in an ecosystem, metatranscriptomics investigates gene expression and therefore provides access to messenger and non-coding RNAs. As the majority of RNA in a cell is composed of ribosomal and transfer RNAs (> 95%), metatranscriptomics typically comprises rRNAs depletion steps to enrich for mRNAs [44]. Metatranscriptomics commonly involves reverse transcription to generate cDNA, which can then be sequenced using the same platforms as for metagenomics [16]. Direct RNA sequencing, bypassing cDNA generation and its associated biases, is also available [45] but has not yet been employed in the context of mixed microbial communities. Although not usually performed in metatranscriptomic studies, 16S and 18S rRNA surveys from RNA samples are recommended prior to metatranscriptome investigations. The SSU rRNA data can then be analysed as indicated above in the context of metagenomics [12], [13], [14]. This can provide some insight into which operational taxonomic units (OTUs) are likely to be active at the time of sampling, information that cannot be deduced from similar data generated at the DNA level. In order to access in situ microbial gene expression metatranscriptomes have to be investigated. Metatranscriptomics offers the unique opportunity to identify novel non-coding RNAs, including small RNAs reported to play key roles in central biological processes such as quorum sensing, stress response and virulence [46], [47], [48]. Shi et al. (2009) detected a large fraction of small RNAs in marine water reportedly involved in the regulation of energy metabolism and nutrient uptake [49]. One of the main challenges of metatranscriptomics is the assembly of non-continuous short-read sequences with uneven sequencing depth due to variation in mRNA abundance within and between microorganisms. In addition, different mRNAs commonly contain repeat patterns, reflecting functional redundancies in proteins, which render the process of assembly even more difficult. Binning and functional annotation strategies similar to those used for metagenomic sequences are employed [17], [18], [19], [20], [21], [22], [23], [24], [25], [26], [27], [28], [29], [30]. Metatranscriptomic data analysis can be considerably facilitated when performed in tandem with metagenomics. Xiong et al. (2012) developed an experimental and analytical pipeline for the analysis of metatranscriptomes in the absence of extended sets of reference genomes [50]. Their workflow employs a peptide-centric search strategy by performing in silico translation of detected transcripts. While Leung et al. (2013) specifically designed a new algorithm for metatranscriptome assembly [51], HUMAnN, which processes unassembled short-read sequences can be used for the analysis of transcribed gene families and pathways and the determination of their corresponding abundance within a microbiome [31]. Interestingly, Desai et al. (2013) developed a computational pipeline (FROMP) to compare metabolic reconstructions from metagenomic and metatranscriptomic datasets [52]. Such comparisons can highlight the discrepancies between metabolic potential and actual transcription, as observed in the case of marine microbial communities [53]. Metatranscriptomics has been successfully employed to investigate the effect of xenobiotics on the human gut microbiota [54]. Indigenous microbial communities were found to respond to xenobiotics by activating drug metabolism, antibiotic resistance and stress response pathways across multiple phyla. This study therefore captured the collateral consequences of xenobiotic treatment. Metatranscriptomics in combination with isotope labelling was also used to decipher the fate of methane and nitrate in anaerobic environments [10]. Impressively, using internal standards for quantitative metagenomics and metatranscriptomics, Satinsky et al. (2014) could suggest different contributions to geochemically relevant processes of free-living and particle-associated microbiota in the Amazon River Plume during a phytoplankton bloom [55]. Particularly, free-living microorganisms were found to express genes involved in carbon, nitrogen and phosphate cycles, while particle-associated microbial communities transcribed genes with relevance to sulphur cycling [55]. The authors, however, recognise the limitations of metatranscriptomics, as mRNA abundance cannot be used as a proxy for microbial activities. In term of ecosystem functioning mRNAs only reflect potential functions since it cannot account for post-transcriptional regulation. Indeed not all mRNAs are translated into proteins and a lack of correlation between mRNA and protein levels has been previously reported [6]. Even though the detection of proteins cannot be strictly correlated with microbial activities and process rates, metaproteomics provides useful insights into microbial functions.

## 4 Metaproteomics: Microbial function

Metaproteomics investigates the proteins (catalytic and structural) collectively expressed within a microbiome and together with metabolomics provides access to ecosystem functioning. The identification of proteins and metabolites can be directly used to construct metabolic models reflecting active pathways and in this context, metaproteomics and metabolomics complement each other very well. Metaproteomics, however, presents some valuable advantages over metabolomics as proteins can be assigned to specific taxa and therefore their detection informs not only on what pathways are active within an ecosystem but also on the identity of species involved in specific functions. In this respect, metaproteomics offers a powerful approach to link community composition to function. The success of metaproteomics is strongly dependent on the availability of relevant genomes to enable high protein identification rates [6]. It is therefore recommended to use metaproteomics in combination with metagenomics, an experimental approach which will result in a synergistic effect since the detection of peptides can assist and validate metagenome annotation [4]. Compared to metagenomics and metatranscriptomics, metaproteomic computational workflows are somewhat less developed [56]. Software tools like MEGAN [57] can be used for metaproteomics, in which case the initial BLAST files are generated directly from protein files, and HUMAnN is also suggested to be amenable for metaproteomic datasets [31]. One of the limitations of MEGAN is that it employs a naïve pathway mapping strategy. Proteins can be involved in more than one biochemical reaction and, consequently, can participate in several metabolic pathways. Also significant in the context of metabolic reconstruction from metagenomic datasets, a naïve pathway mapping strategy (whereby the detection of a protein implies the potential activity of all the biological pathways the protein might be involved in) can lead to an overestimation of the functional diversity of microbial communities. Parsimony approaches, as employed in the HUMAnN pipeline, are then applied to offer a more accurate representation of the functionality of a microbial community by specifically identifying the minimum set of biological pathways that can account for all the protein families detected [31], [58], [59]. While for metagenomics and metatranscriptomics relative quantification and even absolute quantification with the use of internal standards are accessible, protein abundance is harder to determine. In the context of pure-culture proteomics, labelling methods, such as iTRAQ (isobaric tags for relative and absolute quantification) have been developed [60], while in multi-species communities, normalised abundance factors are commonly calculated [61], [62], [63], [64]. The comparison of summer and winter metaproteomes from West Antarctic Peninsula seawaters, using spectral counts for the determination of protein levels, revealed seasonal shifts in abundance of specific taxa through protein assignments, which could be correlated with differences in metabolic activities [65]. Of particular note was the observation that ammonia oxidation was exclusively carried out by archaea during the winter, while bacteria were predominantly involved in this process in the summer. Interestingly, metaproteomics has been used as a tool to compare the physiological states of microbial communities under different environmental conditions [66], [67]. Specifically, the characterisation of the metaproteome from acid mine drainage biofilms grown under laboratory conditions enabled the fine-tuning of the media composition to mimic the natural environment of these microbial communities [66]. Recently, metaproteomics combined with isotope labelling has uncovered a novel family of enzymes involved in hydrocarbon bioremediation [68]. Metaproteomics has also revealed an increasingly important role for a clade of Gammaproteobacterial sulphur oxidizers (SUP05) in marine nutrient cycling in response to climate change [69]. Even though metaproteomics is a powerful tool to link microbial community composition to function, one of the main challenges of metaproteomics is to relate protein abundances to microbial activities, which are ultimately reflected by metabolic fluxes.

## 5 Metabolomics: Microbial activity

Metabolomics is employed to characterise the intermediates and end-products of metabolism. Metabolites are typically of low molecular weights and are mostly in a state of flux, which implies that their compositions and concentrations vary significantly as a function of time within an ecosystem. Metabolomics offers a powerful approach for the characterisation of ecosystem phenotypic traits (at the macro-scale) resulting from the network of interactions occurring between the members of the microbial communities (at the micro-scale). This methodology therefore plays a significant role in determining ecosystem emergent properties and thus is widely used for biomarker discovery and diagnostics [70], [71]. Two experimental workflows can be employed in metabolomics; a targeted approach where known metabolites are quantified and a non-targeted strategy aiming at characterising entire metabolomes [72]. Due to the great variation in metabolite chemical structures, non-targeted metabolomics is commonly characterised by the detection of large fractions of unknown metabolites [73]. In addition, metabolite databases can contain incomplete information and are unsuitable for the identification of isomers [74]. Faecal metabolite profiling of cirrhotic patients revealed the differential detection of 1771 features when compared to control groups. Amongst these, only 16 metabolites could be identified [75]. Despite the low identification rate, liver cirrhosis was shown to correlate with nutrient malabsorption and disruptions in fatty acid metabolism [75]. Over 3500 metabolic features were detected in acid mine drainage biofilms, from which only 56 were identified with more than 90% classified as unknown [76]. Some of these likely represent novel metabolites but this observation was largely attributed to the incompleteness of MS/MS databases. Indeed they are limited to commercially available compounds, which are estimated to represent as little as 50% of all biological metabolites [76]. In this study, metabolomics was combined with isotope labelling, which led to significant improvements in chemical formula prediction particularly for large metabolites [76]. In order to gain some insights into unknown metabolites typically detected in untargeted investigations, modification-specific metabolomics was developed [77]. This novel approach involves the detection of metabolite modification encompassing acetylation, sulfation, glucuronidation, glucosidation and ribose conjugation. The inclusion of modification information to the mass feature during database searches drastically reduces the number of matches for metabolite identification and therefore significantly decreases the time required for this process [77]. Similarly, in order to improve metabolite identification rates in untargeted metabolite profiling, Mitchell et al. (2014) developed an algorithm for the detection of functional groups within metabolite databases [78]. Targeted metabolomics, whereby a pre-determined selection of metabolites are detected and quantified, also constitutes a very valuable experimental approach and has been widely employed in the context of human biology. The monitoring of 158 target metabolites belonging to 25 pathways in serum samples allowed the discrimination between three patient groups [79]. Specifically, 13 and 14 metabolites were identified for the differentiation between colorectal cancer patients from healthy individuals and from polyp patients respectively, thus demonstrating the potential of such an experimental strategy for diagnostics. Targeted metabolite profiling of 212 compounds in blood samples over a period of seven years revealed that over 95% of individuals showed at least 70% of metabotype conservation [80]. In addition over 40% of individuals were uniquely identified by their metabolite profile after seven years. In order to appropriately select relevant metabolites to target, PRMT can be employed when metagenomic sequences are available [33]. The application of PRMT to a time-series bacterial metagenomic dataset from the Western English Channel supported a correlation between bacterial diversity and metabolic capacity of the community [33]. Specific bacterial groups could be linked, for example, to carbohydrate utilisation or total organic nitrogen availability. Importantly, PRMT uncovered some novel biological hypotheses by linking specific taxa to organic phosphate utilization or chitin degradation [33]. Overall the success of metabolomics in the context of mixed microbial communities is limited compared to other omics technologies and importantly the identification of metabolites is not particularly informative in terms of microbial interactions. In order to overcome this limitation and to gain some insights into microbial taxa involved in metabolite production, the combination of metabolomics and metaproteomics can be very useful. Metabolic exchange in an acid mine drainage ecosystem between a dominant protist and the indigenous bacterial community was examined by employing a proteo-metabolomic strategy [81]. The protist was found to selectively secrete organic matter in the environment, which amongst other effects led to a nitrogen bacterial dependence on the protist activities. Even though metabolomics and metaproteomics can be successfully combined to investigate microbial interactions, microbial interrelationships and more specifically microbial cross-feeding can be investigated using stable isotope probing (SIP) techniques.

## 6 Sip-Omics: Microbial interrelationships

Although omics approaches, particularly when used in combination, can provide unparalleled insights into the functioning of mixed microbial communities, specific elemental fluxes and microbial interrelationships cannot be easily uncovered from such datasets. SIP, using for example ^13^C, ^15^N or ^18^O isotope labelling, can be employed to elucidate the fate of specific compounds in complex microbial networks. A drawback of these experimental designs however is the inherent necessity of microcosms or multi-species microbial communities culturing set-ups in laboratory environments, which only approximate in situ conditions. Ideally, isotope labelling should be combined with omics and help tackle specific research questions. In order to verify the activity of a novel pathway, suggested by omics analyses, Ettwig et al. (2010) employed a complex experimental strategy involving the incubation of enrichments cultures with ^13^C labelled methane, ^15^N labelled nitrite and ^18^O labelled nitrite [4]. Haroon et al. (2013) could not only demonstrate, using ^13^C and ^15^N labelling, the anaerobic methane oxidation coupled with nitrate reduction in a novel archaeal lineage but also that the nitrite generated by this pathway was subsequently used by an annamox population. This microbial interrelationship was then further confirmed by the co-localisation of the two microbial taxa [10]. At the DNA and RNA level, isotope labelling has been widely used to capture the identity of the active members of microbial communities involved in the degradation of specific compounds. In this context, labelled and unlabelled microbial fractions are separated by density-gradient centrifugation and SSU rRNA genes are typically amplified [82], [83]. More recently, metagenomic analysis of the separated fractions has been carried out but is mostly limited to targeted approaches as opposed to deep metagenomes. For example, SIP enabled the identification of glycoside hydrolases in metagenomic sequences from labelled fractions of soil microbiota [84]. Targeting the same enzyme families directly from bulk soil resulted in a 3-fold decrease in relative abundance, highlighting the enrichment benefit of combining SIP with targeted metagenomics [85]. SIP was also recently combined with metatranscriptomics. Dumont et al. (2013) analysed metatranscriptomic sequences from both heavy and light fractions after incubating lake sediments with ^13^CH_4_ [86]. While the unlabelled metatranscriptome displayed a wide phylogenetic diversity, the labelled sequences were predominantly assigned to methanotrophs. A high abundance of methane monooxygenase transcripts were detected in labelled datasets, which also provided insights into carbon and nitrogen metabolism [86]. SIP metaproteomics is quite widely used and presents some advantages over RNA-SIP and DNA-SIP. Indeed, labelled and unlabelled protein fractions are not separated and the level of isotope incorporation into amino acids can be measured, which informs on protein turnover and acts as a direct proxy for activity [87]. Furthermore, the limits of detection of heavy labelled isotopes are very low (in the order of 0.1% relative isotope abundance), which allows for i) the use of lower labelled substrate concentrations (closer to in situ conditions) and ii) access to rare taxa [88]. Pan et al. (2011) developed an algorithm to accurately determine ^15^N percentage incorporation into proteins [89]. In this study, isotope labelling was employed to investigate the microbial processes involved in biofilm development and recolonisation. A low protein turnover was observed in the mature biofilm, while the opposite was found in the early stage growth biofilm, reflecting the requirement for de novo protein synthesis in the latter conditions [89]. Protein-SIP was recently employed to investigate the degradation of naphtalene and fluorene in groundwater [90]. Proteins involved in naphtalene metabolism were mostly assigned to *Burkholderiales*, which were strikingly estimated to obtain over 80% of their carbon from the labelled environmental contaminant. Proteins involved in fluorene degradation could not be identified in situ, while *Rhodococcus* was found to play a major role in this process under laboratory conditions [90]. The authors emphasise the significance of this observation, which indicates a biassed enrichment under artificial conditions and a crucial need for in situ investigations to properly examine microbial processes. Some form of metabolomics is always involved in SIP experiments since the detection and concentration of specific labelled metabolites are necessarily investigated. However SIP can also be employed in the context of untargeted metabolomics. Using an elegant experimental strategy comparing unlabelled to labelled substrate metabolic measurements, Hiller et al. (2010) developed a computational method (nontargeted tracer fate detection: NTFD) to quantitatively detect metabolites derived from a specific labelled compound [7]. Combined with other omics, the quantitative NTFD should facilitate the discovery of novel pathways while highlighting metabolic pathway connectivity and microbial interrelationships. SIP metabolomics and metagenomics were recently employed to investigate the microbial anaerobic degradation of cellulose [91]. In this study, labelled and unlabelled fractions were not separated before downstream analyses and only 16S rRNA, 18S rRNA and carbohydrate-binding domain information was extracted from the metagenomic dataset. ^13^C labelled cellulose was found to be mainly degraded by clostridial species and resulted in the production of ^13^C acetic acid and ^13^C propionic acid [91]. Overall SIP represents a very attractive experimental strategy to track down the fate of specific compounds and uncover metabolic pathway connectivity within microbial ecosystems but must be combined with other omics in order to fully exploit its potential.

## 7 Systems biology: Towards microbial ecosystem modelling

Overall, progress in omics technologies is advancing at a fast pace but in order to fully adopt systems biology approaches, omics datasets need to be integrated and to constitute the basis for ecosystem predictive modelling. Furthermore, since the emergent properties of microbial systems are a direct consequence of the network of interactions between the members of the microbial communities and their environment, both physical and microbiological processes need to be considered. Microbial interactions are inherently dependent on temporal and spatial scales and are subject to stochastic processes. To illustrate the importance of spatial organisation, Frey (2010) discusses two scenarios involving the *Escherichia* Col E2 system, in which the outcome of microbial interactions is in direct opposition [92]. The production of the Col E2 toxin by *Escherichia coli* allows the producing strain to kill sensitive competitors but confers a competitive advantage to resistant strains. Indeed, even though resistance has an inherent fitness cost, the toxin-producing strain (resistant to its own toxin) is also bearing the toxin production cost. When grown on agar plates, the three types of strains can coexist, while in agitated liquid medium, only the resistant strains survive [92]. This example highlights the necessity to elucidate the spatial organisation of microbial species within an ecosystem in order to resolve microbial interrelationships. Modelling microbial interactions based on single-species metabolic network reconstruction has led to the prediction of environmental conditions promoting either cooperation or competition between microbial pairs [40], [41], [42], [43], [93], [94], [95]. This kind of strategy typically involves stoichiometric constraint-based modelling using Flux Based Analysis (FBA). In this framework, metabolite fluxes are constrained by mass conservation, thermodynamics (reaction directionality), assumption of steady-state intracellular metabolite concentrations and nutrient availability [96]. These constraints are then used in silico as boundary conditions to find a set of metabolic fluxes that satisfies stoichiometry and maximises a pre-defined biological objective function commonly chosen as biomass production. To refine the prediction of metabolic flux distribution, quantitative proteomics and metabolomics were integrated together with genome-scale metabolic reconstruction [97]. This novel modelling approach was found to predict more accurately (compared to FBA) the metabolic state of human erythrocytes as well as of *E. coli* deletion mutants [97], notably illustrating the versatility of computational methods, applicable to diverse biological contexts. Using dynamic flux balance analysis and stoichiometric models, a novel computational framework, COMETS, could predict the equilibrium species ratio of a three-bacterium community [98]. Interestingly, COMETS can integrate both manually curated and genome-based automated reconstructed stoichiometric models. COMETS is proposed to be scalable to more complex microbial communities [98] and as demonstrated by Yizhak et al. (2010; 97), the integration of other omics could positively impact on COMETS by refining the stoichiometric models employed. Metagenome-based metabolic reconstructions have recently started to emerge, as illustrated by the development of HUMAnN to determine the relative abundance of gene families and pathways from metagenomic datasets [31]. In parallel, comparative metagenomic tools, such as LEfSe (linear discriminant analysis effect size), have been designed specifically for metagenomic biomarker discovery [99]. A very interesting concept in systems-based microbial ecology is the newly developed reverse ecology framework, which aims to translate genomic data into ecological data by predicting the natural environment of a species, including its interactions with other species from genomics [100]. Using this framework, Levy and Borenstein (2013) addressed the forces driving microbial community composition within the human microbiome [101]. They developed a computation framework that could predict co-occurrence patterns from metagenomic datasets, which were verified using experimental observations. Excitingly, they could demonstrate that microbial species composition was predominantly governed by habitat filtering, whereby competitors co-occurred, and not by species assortment. The two patterns, however, are not mutually exclusive. While community composition was found to be mainly dictated by resources for which microorganisms compete, species with complementary requirements were also found to co-exist within microbial communities [101]. Furthermore, Levy and Borenstein (2013) also observed an increase in habitat-filtering signatures within phyla, which indicated that even though phylogenetic closeness can be linked to co-occurrence patterns it cannot solely explain the habitat-filtering dominant structure observed within the human microbiome [101]. Strikingly, mathematical models developed to date in the context of mixed-species microbial communities have only focused on metagenomic datasets [31], [33], [101], [102] while bypassing metatranscriptomics, metaproteomics and metabolomics. These omics methodologies, however, provide valuable insights into ecosystem functioning and, therefore, are imperative for the accurate prediction of ecosystem emergent properties.

## 8 Summary and outlook

The field of omics, along with corresponding computational workflows, is expanding very rapidly and overall a clear move from proof-of-concept studies to real investigations has taken place. A recent breakthrough in metagenomics and metatranscriptomics has been realised with the introduction of internal standards, allowing the corresponding technologies to enter the realm of absolute quantification [55], [103]. Over 10^13^ genes and 10^11^ transcripts were detected per litre of seawater in the Amazon River Plume representing the first quantitative in situ investigation [55]. Carbon and nutrient flux through this natural ecosystem could be resolved and the level of expression of relevant genes was compared in different microenvironments [55]. Tools to accurately quantify protein levels are starting to emerge [63] and this should be followed by the development of adequate internal standard procedures to access absolute quantification, similarly to metagenomics and metatranscriptomics [103]. As discussed above, targeted metabolomics can be powerful in the context of diagnostics [80]. Also, methodologies are being developed to gain some insights into the large fraction of unknown metabolites typically identified in untargeted experimental strategies [77], [78]. Despite the wealth of information that can be derived from omics datasets, pathway connectivity and microbial interrelationships are not easily accessed. This can be partly overcome by combining omics with SIP, which requires a precise experimental setup. Indeed, the use of labelled substrates cannot be performed in natural environments and necessitates laboratory settings, which impose inevitably some artificial constraints resulting in data biases [90]. Therefore, a thorough investigation of the physiological state of microbial communities under laboratory conditions should be carried out and compared to that of their natural habitat prior to SIP, as elegantly demonstrated by Belnap et al. (2010; 66). Datasets obtained from integrated omics approaches can provide unprecedented insights into ecosystem functioning. However, to enable their full exploitation they need to form the basis for mathematical modelling. The concept of reverse ecology and its integration into the computational framework developed by Levy and Borenstein (2013; 101) is a very promising tool to tackle the challenging task of microbial community modelling and constitutes an excellent starting point for the integration of multi-omics datasets. Finally the development of such models will necessitate a true integration of experimental observations and model development with systematic iterative validation and refinement.
